# Persistence of Nasopharyngeal Pneumococcal Vaccine Serotypes and Increase of Nonvaccine Serotypes Among Vaccinated Infants and Their Mothers 5 Years After Introduction of Pneumococcal Conjugate Vaccine 13 in The Gambia

**DOI:** 10.1093/cid/ciy726

**Published:** 2018-08-24

**Authors:** Effua Usuf, Christian Bottomley, Ebrima Bojang, Isatou Cox, Abdoulie Bojang, Rebecca Gladstone, Beate Kampmann, Philip C Hill, Anna Roca

**Affiliations:** 1 Medical Research Council Unit–The Gambia at the London School of Hygiene and Tropical Medicine, Fajara; 2 Department of Infectious Disease Epidemiology, London School of Hygiene and Tropical Medicine, United Kingdom; 3 Sanger Institute, Wellcome Trust, Pathogen Genomics, Cambridge, United Kingdom; 4 The Vaccine Centre, Faculty of Infectious and Tropical Diseases, London School of Hygiene and Tropical Medicine, United Kingdom; 5 Centre for International Health, University of Otago, Otago, New Zealand

**Keywords:** pneumococcal carriage, serotypes, herd-effect, nontypeable

## Abstract

**Background:**

The widespread use of pneumococcal conjugate vaccine (PCV) has brought about a dramatic decrease in pneumococci of vaccine serotypes (VTs) but nonvaccine serotypes (NVTs) have emerged.

**Methods:**

We conducted a cross-sectional survey (CSS) among infants who received 3 doses of 13-valent PCV (PCV13) and their mothers 5 years (CSS3) after PCV13 introduction. Nasopharyngeal swab samples were collected and cultured for isolation of *Streptococcus pneumoniae*. Whole-genome sequencing of the nontypeable strains was performed. Data were compared with those from 2 previous surveys conducted before PCV13 introduction (CSS1) and 1 year later (CSS2).

**Results:**

Among infants, VT carriage decreased from 33.3% (113/339) in CSS1 to 11.4% (40/351) in CSS3 (*P* = .001) while NVTs increased from 53.1% (180/339) in CSS1 to 74.4% (261/351) in CSS3 (*P* < .001). Among mothers, there was a significant decrease in VTs between CSS2 8.4% (29/347) and CSS3 5.6% (19/342) (*P* = .006). NVTs increased from 16.6% (55/331) in CSS1 to 32.2% (110/342) in CSS3 (*P* < .001). In CSS3, the most prevalent VTs were 7F in infants and 3 in mothers, and the most prevalent NVTs were serogroup 16 and nontypeables, respectively. Genomic analysis showed that VTs were more likely than NVTs to lose their ability to express the capsule.

**Conclusions:**

Five years after PCV13 introduction, we show both direct (infants) and indirect effects (mothers) of the vaccine, while NVT replacement has occurred in both groups. Ongoing circulation of VTs warrants further study of their relevance in any consideration of a reduced dose schedule.

## Teaser

Five years after introduction of 13-valent pneumococcal conjugate vaccine (PCV13), pneumococcal carriage has decreased among PCV13-vaccinated infants and their mothers; however, some vaccine serotypes (eg, 3, 7F, and 19A) have persisted and some nonvaccine serotypes (eg, serotype 21 and nontypeable strains) have emerged.

Prevention of pneumococcal disease remains a major global health priority owing to the high disease burden and associated mortality, especially among children in developing countries [1]. A 7-valent vaccine (pneumococcal conjugate vaccine [PCV] 7) was licensed in 2009, but higher-valency formulations (10-valent PCV and 13-valent PCV [PCV13]) have since been licensed [2]. The World Health Organization recommends worldwide PCV introduction, particularly in countries with a mortality rate of >50/1000 live births in children <5 years old [3]. To date, PCVs have been introduced in 59 low- and middle-income countries [4].

The impact of PCVs on pneumococcal disease results from a combination of the direct and indirect effects of the vaccine [5–7]. The latter are a consequence of decreased carriage prevalence among vaccinated individuals and the resulting decrease in pneumococcal transmission within communities [8, 9]. The overall vaccine impact on both disease and carriage, however, has been dampened by an increase in serotypes not included in the vaccine formulations, that is, nonvaccine serotypes (NVTs). This phenomenon is known as serotype replacement [10, 11]. Fortunately, the lower virulence of NVTs means there is nonetheless a net reduction in pneumococcal disease after PCV introduction [12, 13]. In addition to serotype replacement, increased capsular switching (genetic recombination of the proteins on the bacterial capsule) in response to vaccine pressure has been reported [14].

In The Gambia, where PCV7 was introduced in 2009 and replaced by PCV13 in 2011, Mackenzie et al [15] reported an 82% reduction in vaccine serotype (VT) invasive pneumococcal disease (IPD) and a 47% increase in NVT IPD 2 years after PCV13 introduction. These data are consistent with data from 2 successive carriage surveys conducted before PCV13 introduction (2 years after PCV7 and 1 year before PCV13 introduction), which showed a decrease in PCV13 VT carriage in infants but not their mothers (ie, no herd effect). Despite the decrease in VTs, a third of all pneumococcal isolates were VTs [16]. Interestingly, an increase in the prevalence of carriage with nontypeable pneumococci, the majority of which had lost their capsule, was also observed [16].

The increase in NVT carriage and the continued presence of VTs after PCV, both in carriage and disease, are a cause for concern. To continue monitoring the direct and indirect effects of PCV13 among vaccinated infants and their unvaccinated mothers on overall VT and NVT carriage, and to explore vaccine impact on nontypeable strains, we conducted a third pneumococcal carriage survey, 5 years after PCV13 introduction.

## METHODS

### Study Setting

The study was carried out at 2 government health facilities that offer maternal and child health services with regular Expanded Programme on Immunisation clinics. PCV13 was introduced without a catch-up in a 3+0 schedule with doses given at 2, 3 and 4 months. The World Health Organization/United Nations Children’s Fund (UNICEF) immunization data reported >95% coverage of PCV13 dose 3 in 2016 [17].

### Study Design

We conducted a cross-sectional survey (CSS) 5 years after PCV13 in 2016 (CSS3). The results were compared with historical data from 2 earlier surveys conducted before PCV13 introduction in 2011 (CSS1) and 1 year after PCV13 introduction in 2012 (CSS2) [16]. The study design, recruitment sites, entry criteria, sample collection, and laboratory methods were similar, and all 3 surveys were conducted between March and June. In all 3 surveys, we recruited healthy infants 6–12 months of age and their mothers at the Expanded Programme on Immunisation clinics. Infants were recruited only if they had received 3 doses of PCV13, ≥1 month before recruitment and were accompanied by their biological mother. We collected information on demographics and risk factors for carriage by interviewing the mothers. Nasopharyngeal swab samples were collected from the mothers and their infants. Written informed consent was obtained from the mothers. The study was approved by the joint Medical Research Council–Gambia (MRCG) Government Ethics Committee.

### Nasopharygeal Swab Sample Collection

A calcium alginate swab was passed gently down the posterior wall of the nasopharynx. The swab was removed and placed in skim milk–tryptone-glucose-glycerol (STGG) transport medium. The STGG vials were taken to MRCG laboratories, as recommended elsewhere [18, 19].

### Laboratory Processing

The samples were stored at −70°C and processed as described elsewhere for CSS1 and CSS2 [16]. In brief, 200 µL of thawed, vortexed STGG medium was placed into 5 mL of Todd Hewitt broth containing 5% yeast extract, and 1 mL of rabbit serum. This mixture was vortexed and then incubated at 37°C for 4–6 hours. Subsequently, a 50-µL aliquot was inoculated onto gentamycin blood agar and incubated overnight in 5% carbon dioxide at 37°C for the selective isolation of *Streptococcus pneumoniae*. The morphologically distinct alpha hemolytic colonies were screened for optochin susceptibility. Serotyping was performed using latex agglutination technique [20]. As in CSS1 and CSS2, serotyping was repeated for all nontypeable isolates.

### Whole-genome Sequencing for Nontypeable Pneumococcal Serotypes

Twenty-nine nontypeable isolates from CSS1 and CSS2 were previously analyzed using whole-genome sequencing at the Wellcome Sanger Institute. In the current study, we analyzed a further 23 nontypeable isolates obtained from CSS3.

The sequencing, identification of contaminants, and multilocus sequence typing were done as detailed elsewhere [16]. The phylogenetic tree was reconstructed from single-nucleotide polymorphism sites [21] using RAxML software Version 8 [22]. In silico serotype was determined using the k-mer–based serotyping method, SeroBA [23]. This identified serotypes and generated capsule loci gene assemblies. No assemblies were generated for classic nontypeable pneumococci or isolates that have lost their capsule synthesis genes. Assemblies were annotated using Prokka software (Version 2) [24]. To investigate potential reasons for the noncapsule expression, all capsule loci were compared with reference capsule loci derived from Bentley et al [25] using the Artemis comparison tool [26]. Genes showing significant difference to the reference genes were aligned and visualized using SeaView software (Version 4) to investigate for truncations and mutations that may cause loss of function.

### Sample Size Calculation

We targeted 350 infants, for a sample size similar to the those in CSS1 and CSS2. This provided 90% power to detect a 50% reduction in the prevalence of PCV13 VTs and a 25% increase in NVTs compared with CSS2, that, a decrease of PCV13 VT prevalence from 18% to 9% among infants and from 8.1% to 4.1% among mothers, and an increase in PCV13 NVT prevalence from 66.3% to 82.9% among infants and from 16.1% to 20.1% among mothers [16].

### Data Management and Analysis

We calculated PCV7 carriage prevalence (serotypes 4, 6B, 9V, 14, 18C, 19F, and 23F), PCV13 carriage prevalence (PCV7 serotypes +1, 3, 5, 6A, 7F, and 19A), and PCV13 NVT carriage prevalence (all other serotypes, including nontypeables). In addition, we calculated the prevalence of the 6 serotypes contained in PCV13 but not in PCV7. We used Poisson regression with robust standard errors to estimate prevalence ratios, comparing both CSS1 and CSS2 with CSS3, and to adjust for potential confounders. We tested the hypothesis that the proportion of PCV13 VTs was the same among “typeables” (ie, isolates of known serotypes) and nontypeables [16] using Fisher exact test. All analyses were done using Stata 14 software (StataCorp).

## RESULTS

### Characteristics of the Study Participants

We recruited 351 infants and 347 mothers in CSS3. Swab samples were obtained from a total of 1020 mothers and 1040 infants over the 3 surveys (20 mothers had twins). Across the surveys, the median age was 25 years (interquartile range, 21.0–29.3 years) for mothers and 7.9 months (6.8–9.4 months) for infants, and 51.8% of the infants were males. The demographic and epidemiological characteristics of the women and infants were similar across the surveys, except that in CSS1 mothers were less educated and infant antibiotic use was more common (Table 1).

**Table 1.** Characteristics of the Study Participants

Variable	Participants, No. (%)^a^			*P* Value^d^
	CSS1^b,c^ (n = 339)	CSS2^b,c^ (n = 350)	CSS3^c^ (n = 351)	
Era^e^	Before PCV13	1 y after	5 y after	
Health center				
Jammeh Foundation	229 (67.6)	251 (71.7)	250 (71.2)	.43
Sukuta	110 (32.4)	99 (28.3)	101 (28.8)	
Sex^f^				
Male	180 (53.1)	165 (47.3)	195 (55.6)	.07
Female	159 (46.9)	184 (52.7)	156 (44.4)	
Infants age, median (IQR), mo	8.2 (7.0–9.6)	7.6 (6.7–9.2)	8.0 (6.9–9.3)	
Infants age at PCV receipt median (IQR), wk				
Dose 1	10.3 (9.3–11.9)	10.6 (9.3–12.3)	10.0 (9.0–11.6)	…
Dose 2	15.9 (14.1–18.3)	16.6 (14.6–18.7)	15.4 (14.0–17.7)	
Dose 3^g^	21.9 (19.4–24.9)	22.4 (20.1–25.6)	20.7 (18.9–23.4)	
Infant antibiotic treatment^h^				
No	295 (88.6)	319 (92.2)	333 (94.9)	.01
Yes	38 (11.4)	27 (7.8)	18 (5.1)	
Mother’s schooling^i^				
None or <1 y	134 (41.7)	108 (31.3)	135 (39.5)	<.001
1–3 y	44 (13.7)	35 (10.1)	8 (2.3)	
4–6 y	78 (24.3)	44 (12.8)	26 (7.6)	
>6 y	65 (20.3)	158 (45.8)	173 (50.6)	
Mother’s age, median (IQR), y	24.5 (21.0–29.0)	25.0 (21.0–28.0)	25.0 (21.0–30.0)	…
Household size, median (IQR), No. of members	5.0 (4.0–7.0)	3.0 (3.0–5.0)	NA	…

Abbreviations: CSS, cross-sectional survey; IQR, interquartile range; NA, not available (data not collected); PCV, pneumococcal conjugate vaccine; PCV13, 13-valent PCV.

^a^Data represent No. (%) of participants unless otherwise specified.

^b^Data from Roca et al [16].

^**c**^The infants included 20 pairs of twins: 8 CSS1, 3 in CSS2, and 9 in CSS3. There were was 331, 341, and 347 mothers in CSS1, CSS2, and CSS3, respectively.

^**d**^ *P* values obtained with χ^2^ test.

^e^Seven-valent PCV was introduced in 2009, 2 years before the start of this study.

^f^Sex missing for one infant in CSS2.

^g^

Date of receipt of PCV missing for 6 infants in CSS1, 2 in CSS2 and 1 in CSS3.

^h^Antibiotic use within 4 months of survey.

^i^Data on schooling missing for 10 mothers in CSS1 and 2 in CCS2.

### Prevalence of Pneumococcal Carriage Before and 5 Years After PCV13

#### Vaccinated Infants

Overall carriage was approximately 85% over the 3 surveys (Table 2). VT pneumococcal carriage decreased from 33.3% (113 of 339 infants) in CSS1 to 11.4% (40 of 351) in CSS3 (*P* < .001), while NVT carriage increased from 53.1% (180 of 339) in CSS1 to 74.4% (261 of 351) in CSS3 (*P* < .001). VT carriage decreased further between CSS2 and CSS3 (from 18.3% to 11.4%; *P* = .02), while NVT carriage increased (from 66.9% to 74.4%; *P* = .02) (Table 2). For the 6 serotypes included in PCV13 but not in PCV7, there was a significant decrease in prevalence between CSS1 and CSS2 (from 23.9% to 13.7%), with an additional drop to 5.4% in CSS3 (*P* < .001).

**Table 2.** Prevalence of Pneumococcal Carriage in Infants Before (CSS1) and 1 (CSS2) and 5 Years (CSS3) After Introduction of PCV13 Into the Gambian Expanded Programme on Immunisation

Vaccine Group	Prevalence of Carriage, %			CSS2 vs CSS3		CSS1 vs CSS3	
	CSS1 (n = 339)	CSS2 (n = 350)	CSS3 (n = 351)	Adjusted RR^a^ (95% CI)	*P* Value	Adjusted RR^a^ (95% CI)	*P* Value
PCV13 VTs	33.3	18.3	11.4	0.64 (.44–.93)	.02	0.60 (.50–.71)	<.001
PCV13 NVTs	53.1	66.9	74.4	1.12 (1.01–1.23)	.02	1.19 (1.11–1.26)	<.001
PCV13 − 7 VTs	23.9	13.7	5.4	0.40 (.24–.67)	.001	0.23 (.14–.37)	<.001
PCV7 VTs	9.4	4.9	6.0	1.30 (.67–2.53)	.44	0.67 (.31–1.00)	.051
All serotypes	85.8	84.3	85.5	1.02 (.96–1.09)	.46	1.00 (.97–1.04)	.88
PCV13 VTs							
1	0	0	0	NA	NA	NA	NA
3	0	0.3	1.1	3.99 (.45–35.56)	.22	NA	NA
4	0.6	0	0.9	NA	NA	1.20 (.49–2.94)	.68
5	0.3	1.4	0	NA	NA	NA	NA
6A	15.3	5.7	0	NA	NA	NA	NA
6B	0.9	0	0	NA	NA	NA	NA
7F	0	0	3.4	NA	NA	NA	NA
9V	0	0	0.6	NA	NA	NA	NA
14	0.6	0.9	0.6	0.66 (.11–3.96)	.65	0.98 (.37–2.61)	.97
18C	0.6	0	0	NA	NA	NA	NA
19A	8.3	6.3	0.9	0.14 (.04–.45)	.001	0.32 (.18–.58)	<.001
19F	5.6	1.7	2.8	1.66 (.61–4.53)	.32	0.71 (.49–1.04)	.08
23F	1.5	2.3	1.1	0.50 (.15–1.64)	.25	0.88 (.46–1.69)	.70
PCV13 NVTs							
10A	4.7	4	6	1.50 (.77–2.89)	.23	1.13 (.82–1.55)	.46
13	3.5	3.4	3.4	1.00 (.45–2.19)	.99	0.98 (.66–1.46)	.93
15B	8.3	9.1	6.8	0.75 (.45–1.24)	.26	0.91 (.70–1.18)	.48
16	5.9	6.6	9.7	1.47 (.89–2.45)	.14	1.28 (.98–1.67)	.07
19C	0.9	0.3	0	NA	NA	NA	NA
21	3.2	4.9	7.1	1.47 (.81–2.67)	.21	1.48 (1.05–2.10)	.03
23B	1.5	3.1	4.6	1.45 (.68–3.08)	.33	1.76 (1.07–2.89)	.03
34	3.8	2.3	4.6	1.99 (.86–4.60)	.11	1.09 (.76–1.56)	.64
35B	4.7	4.9	4.6	0.94 (.48–1.83)	.85	0.98 (.70–1.38)	.92
NT	0.3	6.0	3.4	0.57 (.28–1.14)	.11	3.40 (1.23–9.42)	.02

Abbreviations: CI, confidence interval; CCS, cross-sectional survey; NA, not applicable; NT, nontypeable; NVTs, nonvaccine serotypes; PCV7, 7-valent pneumococcal conjugate vaccine (PCV); PCV13, 13-valent PCV; PCV13-7, serotypes present in PCV7 but not in PCV13; RR, risk ratio; VTs, vaccine serotypes.

^a^Adjusted for health center, maternal age and education, and infant’s age, sex, and antibiotic intake within 4 weeks of the survey.

Among VTs, 19A decreased in prevalence from 8.3% in CSS1 to 0.9% in CSS3 (*P* < .001), and 19F decreased slightly, from 5.6% to 2.8% (*P* = .08). Serotype 6A decreased from 15.3% in CSS1 to 5.7% in CSS2 (*P* < .001). The most prevalent VT in CSS3 was serotype 7F (3.4%), which had not been seen in the previous surveys (*P* < .001). Serotype 21 was the second most prevalent NVT (7.1%), having increased significantly from CSS1 (3.2%; *P* = .03), and there was also a significant increase in the nontypeables, from 0.3% in CCS1 to 3.4% in CSS3 (*P* = .02).

### Mothers of Vaccinated Infants

Overall pneumococcal carriage increased from 23.0% (76 of 331 mothers) in CSS1 to 37.7% (129 of 342) in CSS3 (*P* < .001) (Table 3). Although VT carriage was similar in all 3 surveys (6%), NVT carriage increased from 16.6% (55 of 331) in CSS1 to 32.2% (110 of 342) in CSS3 (Table 3).

**Table 3.** Prevalence of Pneumococcal Carriage in Mothers Before (CSS1) and 1 Year (CSS2) and 5 Years (CSS3) After Introduction of 13-Valent PCV Into the Gambian Expanded Programme on Immunisation

Vaccine Group	Prevalence of Carriage, %			CSS2 vs CSS3		CSS1 vs CSS3	
	CSS1 (n = 331)	CSS2 (n = 347)	CSS3 (n = 342)	Adjusted RR^a^ (95% CI)	*P* Value	Adjusted RR^a^ (95% CI)	*P* Value
PCV13 VTs	6.6	8.4	5.6	0.58 (.33–1.02)	.06	0.92 (.67–1.27)	.63
PCV13 NVTs	16.6	16.1	32.2	2.01 (1.51–2.67)	<.001	1.40 (1.20–1.63)	<.001
PCV13 − 7 VTs	3.9	6.1	2.3	0.34 (.15–.74)	.007	0.65 (.28,–1.52)	.32
PCV7 VTs	2.7	2.6	3.2	1.14 (.46–2.79)	.78	0.99 (.40-2.48)	.99
All serotypes	23.0	24.2	37.7	1.51 (1.20–1.91)	<.001	1.29 (1.14–1.47)	<.001
PCV13 VTs							
1	0.3	0	0	NA	NA	NA	NA
3	0	0.3	1.8	6.09 (.74–50.38)	.094	NA	NA
4	0.6	0.6	0.6	1.01 (0.14–7.17)	.99	0.98 (.37–2.62)	.97
5	0	0.9	0	NA	NA	NA	NA
6A	1.8	2	0	NA	NA	NA	NA
6B	0	0	0	NA	NA	NA	NA
7F	0	0	0.3	NA	NA	NA	NA
9V	0	0	0.3	NA	NA	NA	Na
14	0.6	0.3	0.6	2.03 (.18–22.31)	.56	0.98 (.37–2.62)	.97
18C	0.3	0.6	0.3	0.51 (.05–5.58)	.58	0.98 (.25–3.93)	.98
19A	1.8	3.2	0.3	0.09 (.01–.71)	.02	0.40 (.14–1.16)	.09
19F	0.6	0.3	1.2	4.06 (.46–36.19)	.21	1.39 (.60–3.24)	.44
23F	0.6	0.9	0.3	0.34 (.04–3.24)	.35	0.70 (.21–2.31)	.55
PCV13 NVTs							
10A	0.6	0.3	2.3	8.12 (1.02–64.65)	.048	1.97 (.91–4.26)	.09
13	0.6	1.7	0.6	0.34 (.07–1.67)	.18	0.98 (.37–2.62)	.97
15B	0.6	1.7	1.2	0.68 (.19–2.38)	.54	1.39 (.60–3.24)	.44
16	2.4	0.6	2.3	4.06 (.87–19.00)	.08	0.98 (.61–1.60)	.95
19C	1.2	0	0	NA		NA	NA
21	0.9	0.9	2.9	3.38 (.94–12.19)	.06	1.80 (.95–3.41)	.07
23B	0	0.9	2.0	2.37 (.62–9.09)	.21	NA	NA
34	1.2	1.2	1.8	1.52 (.43–5.35)	.51	1.20 (.64–2.26)	.56
35B	1.8	0.6	1.8	3.04 (.62–14.99)	.17	0.98 (.56–1.72)	.95
NT	0.3	1.4	3.2	2.23 (.78–6.36)	.13	3.26 (1.17–9.06)	.02

Abbreviations: CSS, cross-sectional survey; NA, not available; NT, nontypeable; NVTs, nonvaccine serotypes; PCV7, 7-valent pneumococcal conjugate vaccine (PCV); PCV13, 13-valent PCV; PCV13-7, serotypes present in PCV7 but not in PCV13; RR, risk ratio; VTs, vaccine serotypes.

^a^Adjusted for health center, maternal age, and maternal education.

Serotype 3 was the most prevalent VT in CSS3 (1.8%), followed by 19F (1.2%). Other VT serotypes that were prevalent in CSS1 and CSS2 decreased considerably; 19A decreased from 1.8% in CSS1 to 0.3% in CSS3, and 6A from 1.8% in CSS1 to 0% in CSS3 (*P* = .01). Several NVTs increased between CSS1 and CSS3. Notably, serotype 21 increased from 0.9% in CSS1 to 2.9% in CSS3 (*P* = .07), and nontypeables increased from 0.3% in CSS1 to 3.2% in CSS3 (*P* = .02) (Table 3).

### Genotypic Analysis of the Nontypeable Serotypes

**Figure 1.**
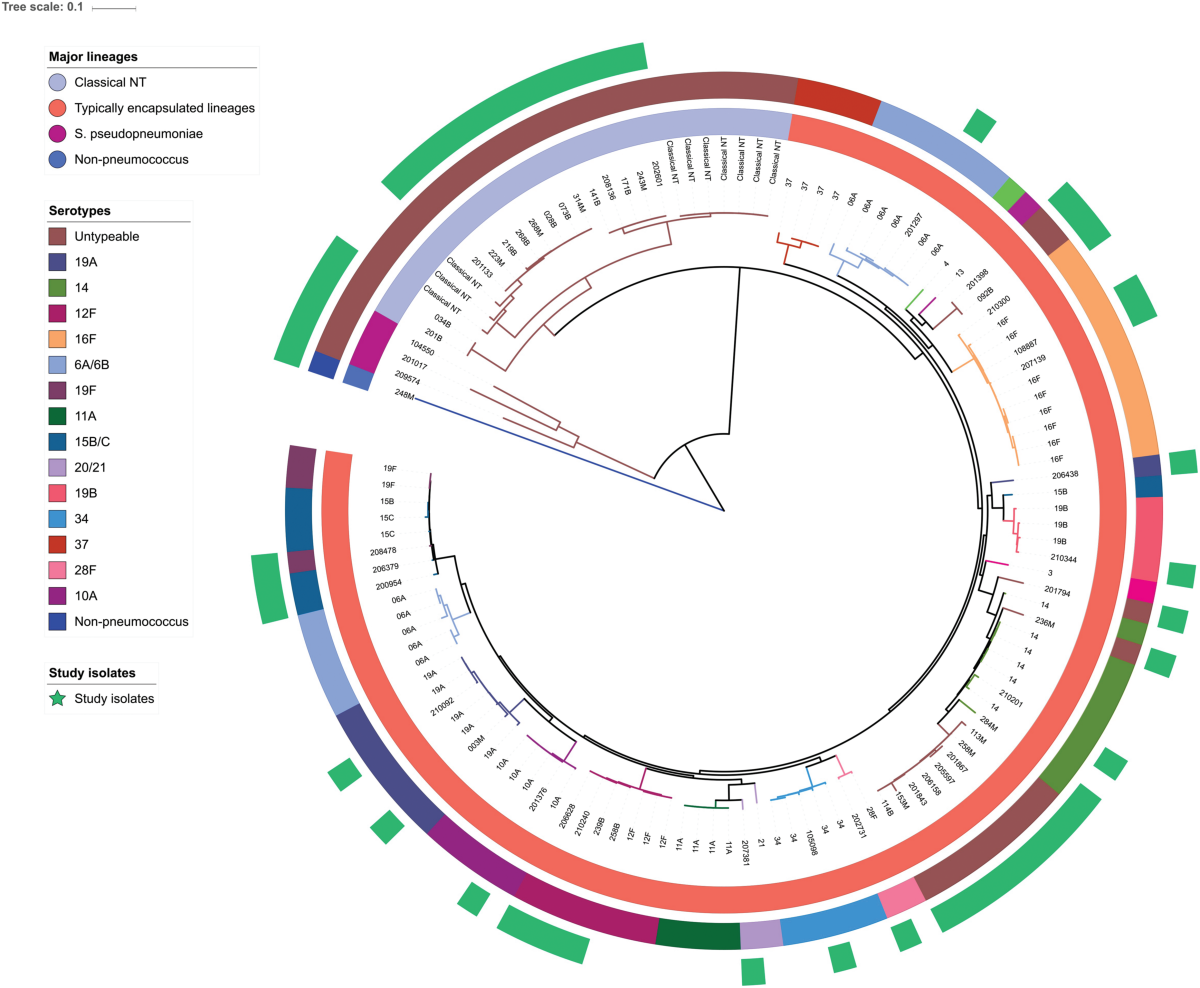
Phylogenetic context of nontypeable isolates. The outer ring includes nontypeable isolates from this study (*green*). The middle ring displays the SeroBA serotype results of all the samples; the brown color represents all isolates without a capsule locus. The leaves are labeled with either the isolate’s serotype (for context samples) or the isolate’s identification number (study samples). The innermost ring depicts the major lineages, classic nontypeables (NT) (*purple*), *Streptococcus pseudopneumoniae* (*red*), and typically encapsulated lineages (*orange*).

A total of 52 phenotypically nontypeable isolates were analyzed, including 3 from CSS1 (2 from mothers and 1 from an infant), 26 from CSS2 (5 from mothers and 21 from infants), and 23 from CSS3 (11 from mothers and 12 from infants). Three isolates were *Streptococccus pseudopneumoniae*—1 from a mother in CSS1 and 2 from infants in CSS2 [16] —and 1 was a nonpneumococcal *Streptococcus* from a mother in CSS3. Fifteen isolates (28.8%) were of the classic nontypeable lineage. The others (33 isolates; 63.5%) were of an encapsulated pneumococcal lineage and had lost their ability to express a capsule through 3 different mechanisms (Figure 1 and Table 4). Eight had a standard capsular polysaccharide synthesis locus but no capsule phenotypically detected, 15 had indels in capsule synthesis genes or complete loss of capsule loci, and 10 had acquired a locus typically found in classically nontypeable isolates.

**Table 4.** Results of Whole-genome Sequencing of Nontypeable Isolates^a^

Isolate No.	Mother or Infant	*S. pneumoniae*, %	*S. pseudopneumoniae,* %	ST	Nearest ST	Capsular Locus Top Hit	Serotype- Specific Sequence Detected	Ancestral Capsular Type From Phylogeny	Conclusion	VT /NVT
CSS1										
108887	Infant	78.08	0.71	3407	…	16F	16F	16F	*wzd* gene loss and truncated *wzh*	NVT
105098	Mother	80.51	0.14	1778	…	34	34	34	Intact capsule synthesis genes; no genetic explanation	NVT
104550	Mother	19.47	40.9	Unknown	…	…	…	…	*S. pseudopneumoniae*	…
CSS2										
201376	Infant	80.46	0.25	5521	…	10A	10A	10A	Intact capsule synthesis genes; no genetic explanation	NVT
207381	Infant	82.09	0.4	Novel ST D	2052	20	20	Long branch, inconclusive	Truncated *whaF* and *wzx* genes	NVT
206628	Infant	81.38	0.08	989	…	12F	12F/A/46	12F/A/46	Intact capsule synthesis genes; no genetic explanation	NVT
210240	Infant	80.96	0.08	989	…	12F	12F/A/46	12F/A/46	Intact capsule synthesis genes; no genetic explanation	NVT
210201	Infant	83.51	0.08	2447	…	14	14	14	Truncated glycosyl transferase genes, *wciY* and *lrp*	VT
201867^b^	Infant	46.64	0.57	Novel ST B	4040	Classic NT	…	14	Capsule switch from 14 to classic NT locus	VT
200954	Mother	81.22	0.25	Novel ST G	975	15B/C	15B/C	15B/C	12-nucleotide insertion at the 5’ end of the initial transferase gene, *wchA*	NVT
206379	Infant	81.51	0.16	4033	…	15B/C	15B/C	15B/C	12-nucleotide insertion at the 5’ end of the initial transferase gene, *wchA*	NVT
207139	Infant	76.55	0.57	3407	…	16F	16F	16F	*wzd* gene loss and truncated *wzh*	NVT
210300	Infant	78.96	0.62	Novel ST F	3407	16F	16F	16F	*wzd* gene loss and truncated *wzh*	NVT
210092	Infant	80.13	0.28	Novel ST E	847	19A	19A	19A	3-nucleotide deletion at the 3” end of the *rmlC* gene	VT
210344	Infant	79.93	0.19	Unknown	7661	19B	…	19B	Intact capsule synthesis genes; no genetic explanation	NVT
206438	Infant	81.8	0.1	202	…	19A	19A	Long branch, inconclusive	3-nucleotide deletion at the 3” end of the rmlC gene	VT
208478	Infant	81.95	0.17	4033	…	19F	19F	19F/15B/C	Intact capsule synthesis genes; no genetic explanation	VT
202731	Infant	81.26	0.08	Novel ST C	6712	28A/F	…	28A/F	6-nucleotide insertion in the *wze* gene	NVT
201297	Mother	81.95	0.34	5734	…	6A/B/C/D	6A/B/C/D	6A	12-nucleotide insertion in 3’ end of the pseudogene *HG262*	VT
201794	Infant	78.82	0.8	Novel ST A	71	38 (Low match; 35%)	…	Long branch, inconclusive	Loss of *cps* locus	…
201398	Mother	76.17	1.3	Novel ST H	3582	Classic NT	…	Long branch, inconclusive	Capsule switch to classic NT locus	…
201843	Infant	77.28	1.11	4040	…	Classic NT	…	14	Capsule switch from 14 to classic NT locus	VT
205597	Infant	75.37	0.96	Novel ST B	4040	Classic NT	…	14	Capsule switch from 14 to classic NT locus	VT
206158	Mother	78.28	0.89	Novel ST B	4040	Classic NT	…	14	Capsule switch from 14 to classic NT locus	VT
201133	Infant	64.27	3.02	344	…	Classic NT	…	Classic NT	Classic NT	…
202601	Mother	65.69	2.64	448	…	Classic NT	…	Classic NT	Classic NT	…
208136	Infant	66.12	2.67	448	…	Classic NT	…	Classic NT	Classic NT	…
201017	Infant	17.54	43.18	Unknown	…	…	…	…	*S. pseudopneumoniae*	…
209574	Infant	20.05	40.36	Unknown	…	…	…	…	*S. pseudopneumoniae*	…
CSS3										
258M	Mother	89.95	1.43	10967	…	Classic NT	…	14	Capsule switch from 14 to classic NT locus	VT
141B	Infant	78.34	3.66	448	…	Classic NT	…	Classic NT	Classic NT	…
219B	Infant	76.67	4.01	344	…	Classic NT	…	Classic NT	Classic NT	…
034B	Infant	77.2	4.86	11729	…	Classic NT	…	Classic NT	Classic NT	…
153M	Mother	83.33	1.86	Novel ST	4040	Classic NT	…	14	Capsule switch from 14 to classic NT locus	VT
114B	Infant	87.33	1.67	4040	…	Classic NT	…	14	Capsule switch from 14 to classic NT locus	VT
223M	Mother	73.83	4.19	344	…	Classic NT	…	Classic NT	Classic NT	…
028B	Infant	77.13	4.1	344	…	Classic NT	…	Classic NT	Classic NT	…
284M	Mother	81.26	0.97	Unknown	…	14	14	14	Truncated *wzg* and *wchA* genes	VT
092B	Infants	88.08	1.84	Novel ST	3582	Classic NT	…	Long branch, inconclusive	Capsule switch to classic NT locus	…
113M	Mother	86.71	1.79	10967	…	Classic NT	…	14	Capsule switch from 14 to classic NT locus	VT
201B	Infant	78.23	4.84	11729	…	Classic NT	…	Classic NT	Classic NT	…
239B	Infant	93.26	0.12	989		12F	12F	12F	No genetic explanation	NVT
248M	Mother	29.05	18.34	Unknown	…	Alternative_aliB_NT	…	Long branch, inconclusive	Nonpneumococcal streptococci	…
236M	Mother	87.4	1.48	Unknown	~908	25F or 25A (Low match; 29.1%)	…	14	…	…
073B	Infant	77.4	4.11	344	…	Classic NT	…	Classic NT	Classic NT	…
268M	Mother	76.62	4.11	344	…	Classic NT	…	Classic NT	Classic NT	…
268B	Infant	76.49	4.14	344	…	Classic NT	…	Classic NT	Classic NT	…
171B	Infant	76.89	3.59	448	…	Classic NT	…	Classic NT	Classic NT	…
003M	Mother	89.41	0.37	847	…	19A	19A	19A	4bp insertion on the wchA. 3bp deletion in the rmlC gene	VT
314M	Mother	77.8	4.16	344	…	Classic NT	…	Classic NT	Classic NT	…
243M	Mother	75.91	3.63	448	…	Classic NT	…	Classic NT	Classic NT	…
258B	Infant	93.29	0.1	989	…	12F	12F	12F	No genetic explanation	NVT

Abbreviations: CSS, cross-sectional survey; NVT, nonvaccine serotype; *S. pneumoniae, Streptococcus pneumoniae*; *S. pseudopneumoniae, Streptococcus pseudopneumoniae*; ST, sequence type; VT, vaccine serotype.

^a^CSS1 and CSS2 updated mechanisms for lack of capsular expression.

^b^Sample contaminated with unclassified organism; pneumococcal coverage was sufficient for analysis and conclusion.

Based on combined data from CSS2 and CSS3, 8 mothers and 19 infants carried nontypeables that were nonclassic; 7 (87.5%) and 8 (42.1%) were of the VT lineage, respectively. Compared with VT carriage prevalence of 22.6% (49 of 217) in mothers and 17.5% (104 of 595) in infants, this corresponds to a 3.9-fold increase (95% confidence interval, 2.7–5.6; *P* < .001) in mothers and a 2.4-fold increase (1.4–4.2; *P* = .01) in infants.

## DISCUSSION

Despite the observed decline in VT pneumococcal prevalence in mothers and infants since the introduction of PCV13 in The Gambia, VT pneumococci continue to circulate in both groups. Among infants, the decrease in PCV13 VT carriage and associated increase in NVTs resulted in no change in overall pneumococcal carriage. In contrast, the decrease in VTs in mothers was less pronounced than the increase in NVTs, resulting in a net increase in overall pneumococcal carriage. Our previous observation that the increase in nontypeable pneumococci after PCV is due mainly to serotypes that have lost their capsule was confirmed by data from CSS3 [16].

Although the direct effect of PCV13 on circulating PCV13 VTs among vaccinated infants was observed 1 year after its introduction (CSS2) [16], 5 years later (CSS3) VTs were still circulating in infants who had received 3 doses of the vaccine. The PCV13 VT prevalence in this study (11.4%) is similar to VT carriage among Gambian newborns 2 years after routine PCV13 [27] but 2-fold higher than PCV13 VT prevalence in Greenlandic children <5 years old (5%) 3 years after PCV13 [28], Belgian children (5.4%) 5 years after PCV13 and 9 years after PCV7 [29], and Australian aboriginal children <5 years old (5.8%) 3 years after PCV13 and 10 years after PCV7 [30]. We saw a significant increase in serotype 19F after PCV13 introduction which was also among the most common VTs after PCV13 in the Belgian study [29]. We also observed an increase in serotype 7F prevalence in CSS3. The increase is surprising because a mathematical model of pneumococcal transmission in The Gambia predicted that PCV13 would eliminate low-transmission VTs, including 7F [31]. A similar increase in 7F was observed in IPD surveillance data after introduction of PCV13 in Australia. However, elsewhere there has been a decline in 7F after PCV13 [32].

Our results suggest a PCV13 herd effect among Gambian mothers of vaccinated infants, particularly for serotypes 6A and 19A; such an effect was not yet apparent 1 year after the introduction of PCV13 (CSS2). However, we have to interpret this decrease in VTs with caution, because the decrease between CSS1 and CSS3 was not significant, and there was an increase between CSS1 and CSS2 driven by the large increase in serotype 19A in CSS2. A cluster-randomized trial conducted in rural Gambia demonstrated a herd effect of PCV [33], as have countries that have introduced PCV7 [34] and countries that have introduced PCV13 with a booster dose [6].

We found that the prevalence of serotype 19A decreased after PCV13 introduction and was low in CSS3 in both mothers and infants. This is in contrast to data from the United Kingdom and Alaska. In the United Kingdom, among the first countries to switch from PCV7 to PCV13, IPD associated with 19A has begun to increase in recent years [35], and in Alaska, PCV13 protection against 19A is waning in older vaccinated children, possibly owing to the emergence of a new genotype. Longer surveillance in our setting is needed to better understand the trend of this serotype in carriage and disease

In mothers, serotype 3 was the most prevalent serotype in CSS3, and cases of IPD caused by this serotype are still seen in The Gambia [15]. This serotype, which had already decreased before PCV13 introduction, shows secular trends in The Gambia [36]. Several carriage and IPD studies have shown that PCV13 has no direct or indirect effect on serotype 3 [37, 38].

Five years after PCV13, we have observed an increase in NVTs in both vaccinated infants and their mothers. For infants, we had already observed an increase between CSS1 and CSS2. In CSS3, we detected a significant increase in serotypes 21 and 23B. These were also the most prevalent serotypes in the post-PCV13 era in studies conducted in The Gambia [27], Italy, and Norway [39, 40]. Unexpectedly, among mothers the increase in NVTs exceeded the decrease in VTs, resulting in an overall increase in carriage. Although this large increase is difficult to explain, it probably reflects an upsurge in pneumococcal transmission rather than biased sampling, because similar field and laboratory methods were used in all 3 surveys and recruitment took place during the same time of the year to minimize any effect of season [41].

Nontypeable strains increased in prevalence over the 3 surveys in both vaccinated infants and mothers, which is consistent with the rise in nontypeable strains causing noninvasive and invasive disease after PCV13 introduction in Taiwan [42]. Further whole-genome sequencing analysis of all the nontypeables confirmed our previous hypothesis that VTs are more likely than NVTs to lose their ability to express the capsule after introduction of PCV. These “capsular switches” may be due to the selection of variants that existed in the pre-PCV era. Capsular loss is relatively common for *S. pneumoniae*, and increased loss due to vaccine pressure has also been reported in other studies [14].

Nontypeable pneumococci are likely to be underreported [43] because they are generally excluded from the analyses in epidemiological studies and vaccine trials, including the ongoing surveillance of the IPD in The Gambia [15]. This may be because their role in disease has been limited. For example, in South Africa over a 10-year period spanning from before to after PCV, and in the United States over a 3-year period in the post-PCV era, nontypeables rarely caused disease [44, 45]. We nonetheless advocate for the inclusion of nontypeable strains in epidemiological surveillance studies owing to their increasing importance and potential for maintaining transmission.

The 3 surveys provide valuable data on the timeliness of vaccination. It is often assumed that vaccination in Africa is substantially delayed and more in line with a 2 + 1 schedule (ie, 2 doses before age 6 months and 1 “booster dose” after 9 months). However, our data suggest that in The Gambia the schedule used is close to 3 + 0 (the median age for PCV dose 3 vaccination was 5 months). Our study therefore represents a genuine evaluation of the 3 + 0 schedule and, as such, contributes to the current debate over the relative merits of the 3 + 0 versus the 2 + 1 PCV schedule [46].

The main limitations of our study were intrinsic to the study design, in that we cannot exclude secular trends in individual serotypes because variability in the prevalence of serotypes was already described before vaccine introduction [47]. In addition, our surveys started only after PCV7 was introduced into routine immunization, and therefore our comparisons were between children vaccinated with PCV7 (CSS1) and those vaccinated with PCV13 (CSS2 and CSS3), capturing only the additional effect of PCV13 over PCV7. There may have been residual confounding, because we did not adjust for the number of siblings, and other studies have shown increased risk of carriage when infants live with other children [27, 47]. However, there was no difference in maternal age between surveys, a potential proxy for number of siblings. Moreover, though we did not collect information on household size in CSS3, there was no association between carriage and household size in CSS1 and CSS2.

In conclusion, we have shown important effects of the introduction of PCV13 into routine immunization in The Gambia, including direct and indirect effects and serotype replacement. Continued disease surveillance and additional carriage surveys are necessary to monitor the persistence of VTs, the emergence of NVTs, and the role of nontypeables in transmission and disease. An alternative vaccination schedule (not necessarily reducing the number of doses) needs to be considered to halt ongoing VT transmission.

## Notes

***Author contributions.*** B. K. and A. R. designed the original studies. E. U., C. B., P. C. H., and A. R. critically reviewed the study proposal and made important contributions throughout the study. I. C. and A. B. led the microbiological isolation of *S. pneumoniae* from the swab samples, and E. B. and R. G. performed the whole-genome sequencing. E. U. coordinated the field work for CSS3, did the analysis, and wrote the first draft of the manuscript. All authors contributed to writing the manuscript and approved the final version.

***Acknowledgments.*** Special thanks to the management of the Jammeh Foundation for Peace and the Sukuta Health Centre, the field staff led by Edrissa Sabally, the junior data manager, Haddy Kanyi, the laboratory technicians, and the mothers and their infants.

***Financial support.*** This work was supported by Medical Research Council–Gambia and the London School of Hygiene and Tropical Medicine (postdoctoral fellowship to E. U.).

***Potential conflicts of interest.*** E. U. served as a consultant for GSK Vaccines Malaria vaccine group (2014–2017). R. G. reports a PhD stipend from Pfizer for studying PCV impact on pneumococcal carriage in the United Kingdom (received 2009–2012). All other authors report no disclosures. All other authors report no potential conflicts. All authors have submitted the ICMJE Form for Disclosure of Potential Conflicts of Interest. Conflicts that the editors consider relevant to the content of the manuscript have been disclosed.

## References

[1] Wahl B, O’Brien KL, Greenbaum A, et al Burden of *Streptococcus pneumoniae* and *Haemophilus influenzae* type b disease in children in the era of conjugate vaccines: global, regional, and national estimates for 2000-15. Lancet Glob Health 2018; 6:e744–57. 29903376 10.1016/S2214-109X(18)30247-X PMC6005122

[2] Esposito S, Tansey S, Thompson A, et al Safety and immunogenicity of a 13-valent pneumococcal conjugate vaccine compared to those of a 7-valent pneumococcal conjugate vaccine given as a three-dose series with routine vaccines in healthy infants and toddlers. Clin Vaccine Immunol 2010; 17:1017–26. 20427630 10.1128/CVI.00062-10 PMC2884425

[3] WHO Publication. Pneumococcal vaccines WHO position paper—2012— recommendations. Vaccine 2012; 30:4717–8. 22621828 10.1016/j.vaccine.2012.04.093

[4] Gavi. Supply and procurement roadmap: pneumococcal vaccine. 2017 Available at: https://www.gavi.org/library/gavi-documents/supply-procurement/pneumococcal-vaccine-roadmap--public-summary/. Accessed 21 May 2018.

[5] Davis SM, Deloria-Knoll M, Kassa HT, O’Brien KL Impact of pneumococcal conjugate vaccines on nasopharyngeal carriage and invasive disease among unvaccinated people: review of evidence on indirect effects. Vaccine 2013; 32:133–45. 23684824 10.1016/j.vaccine.2013.05.005

[6] van Hoek AJ, Sheppard CL, Andrews NJ, et al Pneumococcal carriage in children and adults two years after introduction of the thirteen valent pneumococcal conjugate vaccine in England. Vaccine 2014; 32:4349–55. 24657717 10.1016/j.vaccine.2014.03.017

[7] Sigurdsson S, Erlendsdóttir H, Quirk SJ, et al Pneumococcal vaccination: direct and herd effect on carriage of vaccine types and antibiotic resistance in Icelandic children. Vaccine 2017; 35:5242–8. 28823621 10.1016/j.vaccine.2017.08.020

[8] Hammitt LL, Bruden DL, Butler JC, et al Indirect effect of conjugate vaccine on adult carriage of *Streptococcus pneumoniae*: an explanation of trends in invasive pneumococcal disease. J Infect Dis 2006; 193:1487–94. 16652275 10.1086/503805

[9] Miller E, Andrews NJ, Waight PA, Slack MP, George RC Herd immunity and serotype replacement 4 years after seven-valent pneumococcal conjugate vaccination in England and Wales: an observational cohort study. Lancet Infect Dis 2011; 11:760–8. 21621466 10.1016/S1473-3099(11)70090-1

[10] Weinberger DM, Malley R, Lipsitch M Serotype replacement in disease after pneumococcal vaccination. Lancet 2011; 378:1962–73. 21492929 10.1016/S0140-6736(10)62225-8 PMC3256741

[11] Feikin DR, Kagucia EW, Loo JD, et al; Serotype Replacement Study Group Serotype-specific changes in invasive pneumococcal disease after pneumococcal conjugate vaccine introduction: a pooled analysis of multiple surveillance sites. PLoS Med 2013; 10:e1001517. 24086113 10.1371/journal.pmed.1001517 PMC3782411

[12] Whitney CG, Farley MM, Hadler J, et al; Active Bacterial Core Surveillance of the Emerging Infections Program Network Decline in invasive pneumococcal disease after the introduction of protein-polysaccharide conjugate vaccine. N Engl J Med 2003; 348:1737–46. 12724479 10.1056/NEJMoa022823

[13] Gladstone RA, Jefferies JM, Tocheva AS, et al Five winters of pneumococcal serotype replacement in UK carriage following PCV introduction. Vaccine 2015; 33:2015–21. 25776920 10.1016/j.vaccine.2015.03.012 PMC4392391

[14] Croucher NJ, Kagedan L, Thompson CM, et al Selective and genetic constraints on pneumococcal serotype switching. PLoS Genet 2015; 11:e1005095. 25826208 10.1371/journal.pgen.1005095 PMC4380333

[15] Mackenzie GA, Hill PC, Jeffries DJ, et al Effect of the introduction of pneumococcal conjugate vaccination on invasive pneumococcal disease in The Gambia: a population-based surveillance study. Lancet Infect Dis 2016; 16:703–11. 26897105 10.1016/S1473-3099(16)00054-2 PMC4909992

[16] Roca A, Bojang A, Bottomley C, et al; Pneumo13 Study Group Effect on nasopharyngeal pneumococcal carriage of replacing PCV7 with PCV13 in the Expanded Programme of Immunization in The Gambia. Vaccine 2015; 33:7144–51. 26592141 10.1016/j.vaccine.2015.11.012 PMC5352730

[17] World Health Organization. WHO vaccine-preventable diseases: monitoring system. 2018 global summary: WHO UNICEF estimates time series for Gambia. Geneva, Switzerland: World Health Organization Available at: http://apps.who.int/immunization_monitoring/globalsummary/estimates?c=GMB. Accessed 15 July 2018.

[18] O’Brien KL, Nohynek H; World Health Organization Pneumococcal Vaccine Trials Carriage Working Group Report from a WHO Working Group: standard method for detecting upper respiratory carriage of *Streptococcus pneumoniae*. Pediatr Infect Dis J 2003; 22:e1–11. 10.1097/01.inf.0000049347.42983.77 12586987

[19] Satzke C, Turner P, Virolainen-Julkunen A, et al; WHO Pneumococcal Carriage Working Group Standard method for detecting upper respiratory carriage of *Streptococcus pneumoniae*: updated recommendations from the World Health Organization Pneumococcal Carriage Working Group. Vaccine 2013; 32:165–79. 24331112 10.1016/j.vaccine.2013.08.062

[20] Porter BD, Ortika BD, Satzke C Capsular serotyping of *Streptococcus pneumoniae* by latex agglutination. J Vis Exp 2014 :51747. 25285991 10.3791/51747 PMC4828145

[21] Page AJ, Taylor B, Delaney AJ, et al SNP-sites: rapid efficient extraction of SNPs from multi-FASTA alignments. Microb Genom 2016; 2:e000056. 28348851 10.1099/mgen.0.000056 PMC5320690

[22] Stamatakis A RAxML version 8: a tool for phylogenetic analysis and post-analysis of large phylogenies. Bioinformatics 2014; 30:1312–3. 24451623 10.1093/bioinformatics/btu033 PMC3998144

[23] Epping L, van Tonder A, Gladstone R, et al SeroBA: rapid high-throughput serotyping of *Streptococcus pneumoniae* from whole genome sequence data. Microb Genom. 2018; 4. doi:10.1099/mgen.0.000186 10.1099/mgen.0.000186 PMC6113868 29870330

[24] Seemann T Prokka: rapid prokaryotic genome annotation. Bioinformatics 2014; 30:2068–9.24642063

[25] Bentley SD, Aanensen DM, Mavroidi A, et al Genetic analysis of the capsular biosynthetic locus from all 90 pneumococcal serotypes. PLoS Genet 2006; 2:e31. 16532061 10.1371/journal.pgen.0020031 PMC1391919

[26] Carver TJ, Rutherford KM, Berriman M, Rajandream MA, Barrell BG, Parkhill J ACT: the Artemis comparison tool. Bioinformatics 2005; 21:3422–3. 15976072 10.1093/bioinformatics/bti553

[27] Usuf E, Bojang A, Camara B, et al Maternal pneumococcal nasopharyngeal carriage and risk factors for neonatal carriage after the introduction of pneumococcal conjugate vaccines in The Gambia. Clin Microbiol Infect 2018; 24:389–95. 28743545 10.1016/j.cmi.2017.07.018

[28] Navne JE, Koch A, Slotved HC, et al Effect of the 13-valent pneumococcal conjugate vaccine on nasopharyngeal carriage by respiratory pathogens among Greenlandic children. Int J Circumpolar Health 2017; 76:1309504. 28467237 10.1080/22423982.2017.1309504 PMC5497538

[29] Wouters I, Van Heirstraeten L, Desmet S, et al; NPcarriage Study Group Nasopharyngeal *S. pneumoniae* carriage and density in Belgian infants after 9 years of pneumococcal conjugate vaccine programme. Vaccine 2018; 36:15–22. 29180027 10.1016/j.vaccine.2017.11.052

[30] Collins DA, Hoskins A, Snelling T, et al Predictors of pneumococcal carriage and the effect of the 13-valent pneumococcal conjugate vaccination in the Western Australian aboriginal population. Pneumonia (Nathan) 2017; 9:14. 29021946 10.1186/s41479-017-0038-x PMC5611608

[31] Bottomley C, Roca A, Hill PC, Greenwood B, Isham V A mathematical model of serotype replacement in pneumococcal carriage following vaccination. J R Soc Interface 2013; 10:20130786. 24132203 10.1098/rsif.2013.0786 PMC3808555

[32] Horacio AN, Silva-Costa C, Lopes JP, Ramirez M, Melo-Cristino J; Portuguese Group for the Study of Streptococcal I Serotype 3 remains the leading cause of invasive pneumococcal disease in adults in Portugal (2012–2014) despite continued reductions in other 13-valent conjugate vaccine serotypes. Front Microbiol 2016; 7:1616. 27790208 10.3389/fmicb.2016.01616 PMC5064670

[33] Roca A, Dione MM, Bojang A, et al Nasopharyngeal carriage of pneumococci four years after community-wide vaccination with PCV-7 in The Gambia: long-term evaluation of a cluster randomized trial. PLoS One 2013; 8:e72198. 24086259 10.1371/journal.pone.0072198 PMC3785494

[34] Loo JD, Conklin L, Fleming-Dutra KE, et al Systematic review of the indirect effect of pneumococcal conjugate vaccine dosing schedules on pneumococcal disease and colonization. Pediatr Infect Dis J 2014; 33(suppl 2):S161–71. 24336058 10.1097/INF.0000000000000084 PMC3940524

[35] Ladhani SN, Collins S, Djennad A, et al Rapid increase in non-vaccine serotypes causing invasive pneumococcal disease in England and Wales, 2000-17: a prospective national observational cohort study. Lancet Infect Dis 2018; 18:441–51. 29395999 10.1016/S1473-3099(18)30052-5

[36] Roca A, Hill PC, Townend J, et al Effects of community-wide vaccination with PCV-7 on pneumococcal nasopharyngeal carriage in the Gambia: a cluster-randomized trial. PLoS Med 2011; 8:e1001107. 22028630 10.1371/journal.pmed.1001107 PMC3196470

[37] Waight PA, Andrews NJ, Ladhani NJ, Sheppard CL, Slack MP, Miller E Effect of the 13-valent pneumococcal conjugate vaccine on invasive pneumococcal disease in England and Wales 4 years after its introduction: an observational cohort study. Lancet Infect Dis 2015; 15:629. 10.1016/S1473-3099(15)00028-6 PMC7130081 26008826

[38] Moore MR, Link-Gelles R, Schaffner W, et al Effect of use of 13-valent pneumococcal conjugate vaccine in children on invasive pneumococcal disease in children and adults in the USA: analysis of multisite, population-based surveillance. Lancet Infect Dis 2015; 15:301–9. 25656600 10.1016/S1473-3099(14)71081-3 PMC4876855

[39] Zuccotti G, Mameli C, Daprai L, et al; PneuMi Study Group (PMSG) Serotype distribution and antimicrobial susceptibilities of nasopharyngeal isolates of *Streptococcus pneumoniae* from healthy children in the 13-valent pneumococcal conjugate vaccine era. Vaccine 2014; 32:527–34. 24342249 10.1016/j.vaccine.2013.12.003

[40] Steens A, Caugant DA, Aaberge IS, Vestrheim DF Decreased carriage and genetic shifts in the *Streptococcus pneumoniae* population after changing the seven-valent to the thirteen-valent pneumococcal vaccine in Norway. Pediatr Infect Dis J 2015; 34:875–83. 26020410 10.1097/INF.0000000000000751

[41] Bojang A, Jafali J, Egere UE, et al Seasonality of pneumococcal nasopharyngeal carriage in rural Gambia determined within the context of a cluster randomized pneumococcal vaccine trial. PLoS One 2015; 10:e0129649. 26132206 10.1371/journal.pone.0129649 PMC4488590

[42] Chen HH, Hsu MH, Wu TL, et al Non-typeable *Streptococcus pneumoniae* infection in a medical center in Taiwan after wide use of pneumococcal conjugate vaccine. J Microbiol Immunol Infect 2018; doi:10.1016/j.jmii.2018.04.001. [Epub ahead of print]. 10.1016/j.jmii.2018.04.001 29804657

[43] Keller LE, Robinson DA, McDaniel LS Nonencapsulated *Streptococcus pneumoniae*: emergence and Pathogenesis. MBio 2016; 7:e01792. 27006456 10.1128/mBio.01792-15 PMC4807366

[44] Park IH, Geno KA, Sherwood LK, Nahm MH, Beall B Population-based analysis of invasive nontypeable pneumococci reveals that most have defective capsule synthesis genes. PLoS One 2014; 9:e97825. 24831650 10.1371/journal.pone.0097825 PMC4022640

[45] Mohale T, Wolter N, Allam M, et al Genomic analysis of nontypeable pneumococci causing invasive pneumococcal disease in South Africa, 2003–2013. BMC Genom 2016; 17:470. 10.1186/s12864-016-2808-x PMC4928513 27334470

[46] Whitney CG Examining duration of protection: should a booster dose be part of all infant pneumococcal conjugate vaccine programs?Clin Infect Dis 2018; 67:375–7. 29471317 10.1093/cid/ciy135

[47] Hill PC, Cheung YB, Akisanya A, et al Nasopharyngeal carriage of *Streptococcus pneumoniae* in Gambian infants: a longitudinal study. Clin Infect Dis 2008; 46:807–14. 18279039 10.1086/528688

